# Overall health and drinking behavior among pregnant and breastfeeding women in Korea

**DOI:** 10.4178/epih.e2019036

**Published:** 2019-09-01

**Authors:** Eun Gyeong Kim

**Affiliations:** Department of Nursing, Kunsan National University, Gunsan, Korea

**Keywords:** Pregnant, Breastfeeding, Alcohol, Health status, Korea

## Abstract

**OBJECTIVES:**

The study was to conduct a comparative assessment of drinking behaviors and overall health among pregnant and breastfeeding women.

**METHODS:**

This study used data collected from the Korean Community Health Survey in 2015. Data obtained from 2,156 pregnant or breastfeeding women were analyzed using descriptive statistics, the t-test, the chi-square test, and the Pearson correlation coefficient.

**RESULTS:**

Current drinking and alcohol consumption were higher among pregnant women than among breastfeeding women. Depression was twice as common among breastfeeding women than among pregnant women, and stress was much higher among breastfeeding women as well. Breastfeeding women also had lower subjective dental health and more unmet medical needs than pregnant women.

**CONCLUSIONS:**

Although pregnant women were in better overall health than breastfeeding women, many of them were unable to stop drinking, which is a risky and adverse health behavior that negatively affects maternal and fetal health. In order to reduce drinking among pregnant and breastfeeding women, it is necessary to develop a tailored, standardized educational program and national guidelines.

## INTRODUCTION

The main agenda for Korean maternal and child health in the 21st century pertains not only to changes in the social environment, such as low fertility and advanced maternal age, but also to threats to maternal and child health due to unhealthy lifestyles, such as smoking and drinking.

As women’s social activities have increased and the phenomenon of non-marriage or late marriage has become more widespread, it is increasingly emphasized that measures should be taken to improve the health of future generations [1]. Optimizing maternal health during pregnancy, childbirth, and the postnatal period is essential for achieving healthy outcomes for both the mother and the fetus [2]. Investments in health during the fetal period and infancy can improve the chances of healthy outcomes early in life and build a foundation for a lifetime of good health [3]. For these reasons, maternal health should be seen as the foundation for lifetime health and carries important health implications across generations.

Previous studies have reported that factors affecting maternal and child health include smoking, drinking, exercise, and sleeping behavior during pregnancy and breastfeeding [4-7]. Use of alcohol during the perinatal period can lead to adverse outcomes of pregnancy, including fetal alcohol syndrome (FAS), low birth weight, stillbirth, spontaneous abortion, and prematurity [7]. According to a comprehensive systematic literature search on the prevalence of binge drinking during pregnancy among the general population by country and World Health Organization (WHO) region, the drinking rate was 20.0% among pregnant women in Korea, and the binge drinking rate during pregnancy was 3.7% [8]. The 2014 WHO guidelines prioritize preventing, reducing, and ceasing perinatal alcohol consumption as essential components of optimizing the health and well-being of mothers and children [7]. According to the Behavioral Risk Factor Surveillance System surveys, the monthly drinking rate during the breastfeeding period for the first 3 months after childbirth was 54% [9]. As drinking becomes more common among women, programs to prevent drinking during pregnancy and breastfeeding must be strengthened.

Some women feel stressed and anxious as they experience both physical and psychological changes during pregnancy [10,11]. When pregnant women feel depressed or stressed, it not only damages their physical and mental health, but also affects attachment formation with the fetus and fetal weight [11,12].

Consequently, health behaviors and conditions such as drinking, stress, and depression need to be managed to improve the health of mothers and fetuses. In particular, as Korea faces a low birthrate, the need to improve maternal health has been increasingly emphasized. To develop a strategy for improving maternal health, advance surveillance should be implemented. However, domestic surveys collected from pregnant and breastfeeding women regarding their current health conditions have been insufficient. Most such surveys have been conducted among specific groups, and it is difficult to generalize those survey results to a national level. The basic and most important task in developing policy measures for pregnant and breastfeeding women at a national level is to verify their current condition by utilizing nationally representative data. Therefore, this study aimed to analyze the current status of pregnant women and breastfeeding mothers and to utilize the results as preliminary data for developing maternal and child health programs.

## MATERIALS AND METHODS

### Data source and sample

This study constituted a secondary analysis that aimed to examine pregnant and breastfeeding women’s drinking and health conditions. Data from the Korean Community Health Survey (KCHS) 2015 were analyzed. At the selected sample points, households were selected through systematic sampling, and all adults aged 19 and older in the selected households participated in the KCHS. There were a total of 228,558 participants, and among them, data from 2,156 pregnant or breastfeeding women were used for the final analysis in this study.

### Measures

#### Socio-demographic characteristics

Age, education level, and occupation were analyzed as socio-demographic characteristics. Education level was measured on an 8-point Likert-type scale, with higher scores indicating higher levels of education (1=none, 8=graduate school or higher).

#### Current drinking status

Current drinking status was measured using the following variables: current drinking, alcohol consumption frequency, alcohol consumption volume, and high-risk drinking. Current drinking was defined as drinking alcohol at least once per month in the current year. Alcohol consumption frequency was measured on a 5-point scale, with higher scores indicating a higher alcohol consumption frequency. Alcohol consumption volume was measured on a 5-point scale, with higher scores indicating higher alcohol consumption volume. Those who responded that they drank at least 5 glasses of alcohol at least twice a week were categorized into the high-risk alcohol consumption group according to the definition of high-risk drinking for women in the Korean National Health and Nutrition Examination Survey 2014.

#### Health status

Health status consisted of depression, stress, subjective health status, subjective dental health, sleep duration, and unmet medical care needs. For depression, participants responded “yes” or “no” to a question asking whether they had felt sad or depressed enough to affect their daily life for more than 2 consecutive weeks in the past year. Stress was measured on a 4-point scale, with higher scores indicating higher levels of stress. Both subjective health and subjective dental health were measured on a 5-point scale, with higher scores indicating better health. Sleep duration was measured by the question, “How many hours do you sleep in a day?” and unmet medical care needs were assessed using the question “During the past year, was there ever a time when you wanted medical care but could not get it?”, with a response of either “yes” or “no.”

### Statistical analysis

SPSS version 21.0 (IBM Corp., Armonk, NY, USA) was used for the data analysis. Differences in participants’ characteristics were analyzed using the t-test and chi-square test, as appropriate. Correlations between the variables were analyzed using the Pearson correlation coefficient.

### Ethics statement

The raw data were requested from the KCHS homepage (http://chs.cdc.go.kr/), and were obtained with all private information remaining anonymous.

## RESULTS

### Current drinking and health status in relation to pregnancy and breastfeeding

Among the 2,156 participants, 1,194 were pregnant and 962 were breastfeeding. The differences in current drinking and health status between pregnant and breastfeeding women were as follows (Table 1). Age (t=-2.69, p=0.007) and education level (t=-2.56, p=0.010) were significantly higher among breastfeeding women than among pregnant women, and more pregnant women than breastfeeding women reported having an occupation (χ^²^=83.30, p<0.001). Current drinking (χ^²^=159.33, p<0.001) and alcohol consumption volume (t=4.45, p<0.001) were significantly higher among pregnant women. Depression was significantly more common among breastfeeding women (χ^²^=13.88, p<0.001), and their stress levels were higher (t=-7.58, p<0.001). Subjective health status was higher among pregnant women, but the difference was not statistically significant. Subjective dental health (t=3.02, p=0.003) was higher and sleep duration (t=18.12, p<0.001) was longer among pregnant women, and the results were statistically significant. The proportion of participants with unmet medical needs was 16.2% (10.3% for pregnant women and 23.6% for breastfeeding women). Unmet medical needs were significantly more common among breastfeeding women (t=69.25, p<0.001).

### Relationships among variables

Among pregnant women, there were statistically positive correlations between subjective health and subjective dental health (r=0.27, p<0.001), and subjective health and sleep duration (r=0.08, p<0.001). By contrast, for the same group, there were statistically significant negative correlations between stress and subjective health (r=-0.24, p<0.001), stress and subjective dental health (r=-0.12, p<0.001), and stress and sleep duration (r=-0.16, p<0.001). Stress (t=-5.47, p<0.001), subjective health status (t=2.13, p<0.05), and subjective dental health (t=3.11, p<0.05) were significantly related to depression. Stress (t=-4.79, p<0.001), subjective health status (t=2.43, p<0.05), and subjective dental health (t=4.16, p<0.001) were significantly related to unmet medical needs. There was a statistically significant relationship between depression and unmet medical needs (χ^²^=9.14, p<0.05) (Table 2).

Among breastfeeding women, there were statistically positive correlations between subjective health and subjective dental health (r=0.23, p<0.001), and subjective health and sleep duration (r=0.07, p<0.05). For the same group, the results showed statistically significant negative correlations between stress and subjective health (r=-0.21, p<0.001), stress and subjective dental health (r=-0.12, p<0.001), and stress and sleep duration (r=-0.18, p<0.001). Stress (t=-7.07, p<0.001) and subjective health status (t=3.69, p<0.001) were significantly related to depression. Stress (t=-6.62, p<0.001), subjective health status (t=5.26, p<0.001), and subjective dental health (t=5.26, p<0.001) were significantly related to unmet medical needs. There was a statistically significant relationship between depression and unmet medical needs (χ^²^=33.66, p<0.001) (Table 2).

## DISCUSSION

This study was conducted to verify the current drinking and health status of pregnant and breastfeeding women and to prepare preliminary data for maternal health promotion programs. Current drinking was more common and alcohol consumption was higher among pregnant women than among breastfeeding women, possibly because pregnant women are not aware of their pregnancy status during early pregnancy. For pregnant women, 40.8% reported current drinking. This figure was lower than 50.2%, the rate of women who reported drinking during pregnancy in the study of Yeom [13], but higher than 30.3%, the rate of American women who reported drinking during pregnancy [14]. Pregnant women’s alcohol consumption is the major cause of FAS and fetal alcohol spectrum disorder, and causes other serious problems. If alcohol metabolites accumulate in the fetal brain, the baby may be born with a permanent disability due to brain damage [15]. Among breastfeeding women, 30.9% reported current drinking. Alcohol has negative effects on infants through human milk and it is recommended that women who drink avoid breastfeeding for at least 1 day in order to prevent damage to the infant [16]. Consequently, more efforts should be made to prevent alcohol consumption during pregnancy and breastfeeding and to provide systematic education about the risks of alcohol consumption during pregnancy and breastfeeding, as well as the need to quit drinking. In the USA, activities to prevent FAS through ongoing research, monitoring, and tracking are performed through the annual National Survey on Drug Use and Health, but in Korea, insufficient ongoing research and interventions have been implemented to prevent alcohol consumption during pregnancy. A previous study [12] reported that working women sometimes consumed alcohol when dining with colleagues from work because they could not yet announce their pregnancy. To prevent alcohol consumption during pregnancy, not only individuals and families need to be considered, but also the broader sociocultural system.

The results of this study show that depression was twice as common among breastfeeding women than among pregnant women and that stress was also higher among breastfeeding women. Postpartum depression, which is experienced by many women, can lead to negative results regarding women’s maternal role and negative effects on the whole family. For this reason, depression prevention and interventions to address depression are very important [17]. The results of the correlation analysis showed that stress was significantly negatively correlated with subjective health, subjective dental health, and sleep duration among pregnant and breastfeeding women. Researchers have reported that mental health problems such as stress and depression are related to periodontal diseases [18] and that mental health problems affect both women’s health and fetal health because cortisol released due to stress can be delivered to the fetus through the placenta, causing neurological problems in the fetus [19]. It has also been proven that pregnant women’s mental health problems are relevant to a wide range of adverse fetal health effects, such as low birth weight, mental disorders, and abnormal behavior [20].

The study by Song [17] found that among primiparous mothers with insufficient knowledge and experience to take care of newborn infants, lower subjective health led to more serious parenting stress. That reported result is in line with the finding of this study that among breastfeeding women, stress was negatively correlated with subjective health. Therefore, classes or education for pregnant women need to involve methods to help breastfeeding women to learn about causes of stress and to build their competence for managing and overcoming stress through adaptive patterns such as emotional regulation and positive thinking.

With regard to average sleep duration, pregnant women averaged approximately 7.5 hours of sleep per night, and breastfeeding women averaged approximately 6.5 hours, indicating that breastfeeding women slept for fewer hours. This result is in line with the finding of Kim & Park [21] that the average sleep duration of 8.1 hours per night during pregnancy dropped to 5.8 hours after childbirth. Previous studies found that women who had recently given birth had lower sleep quality than women in general due to a reduction in their total sleep duration, an increase in sleep fragmentation and less restorative sleep [22] and that lower sleep quality due to these factors worsened postnatal depression [4]. Kempler et al. [23] reported that education given to pregnant women helped improve the quality of sleep after childbirth and reduce symptoms of postpartum depression. Consequently, in Korea, research on interventions and methods to improve sleep quality after childbirth needs to be conducted.

Breastfeeding women had lower subjective dental health than pregnant women. Stress was higher and depression was more common among breastfeeding women, and stress was negatively correlated with subjective dental health. According to a previous study, psychological factors such as stress and depression cause physiological changes to physical functions such as the nervous system, endocrine system, and immune system; increase the incidence of infections by raising the levels of C-reactive protein, interleukin (IL)-1, and IL-6; and increase the incidence of periodontal diseases and tooth loss by destroying periodontal tissues [18]. From these findings, it can be deduced that breastfeeding women had lower subjective dental health than pregnant women because they had higher levels of stress and depression. A comprehensive intervention program to promote dental care behaviors needs to be developed in consideration of various health-related characteristics of breastfeeding women, such as psychological stress and pregnancy conditions.

A limitation of this study is that it is based on self-report surveys in which participants self-assessed their situation. Most people tend to underreport their own drinking; thus, it should be considered that the study participants might have underreported their drinking to an even greater extent because it is known that alcohol consumption during pregnancy or breastfeeding is harmful to health. Another limitation is that this research utilized a cross-sectional study design, making it impossible to infer causal relationships. Because there are insufficient studies on pregnant women and only a few studies on breastfeeding women, this study has significance for research on the actual conditions of pregnant and breastfeeding women’s drinking and health.

This study was conducted to examine the current drinking and health status of pregnant and breastfeeding women and to prepare data that can be utilized as a basis for promoting women’s health. The results of this study showed that current drinking was more common and alcohol consumption was higher among pregnant women, while depression was more common and stress was higher among breastfeeding women. Although pregnant women were in better overall health than breastfeeding women, many of them could not quit drinking, which is an undesirable health behavior. Because healthy lifestyles are important even before pregnancy, programs for healthy lifestyle management are required as early as adolescence, and a variety of self-support programs to maintain positive mental and psychological behaviors should be developed from a lifespan perspective. To provide customized health education for pregnant women and breastfeeding women, education programs need to be standardized, and national level guidelines should be developed.

## Figures and Tables

**Table 1.** Current drinking and health status in relation to pregnancy and breastfeeding (n=2,156)

Variables		Pregnant	Breastfeeding	χ² or t	p-value
Age (yr)		31.64±4.26	32.12±4.00	-2.69	0.007
Education		6.34±0.92	6.44±0.89	-2.56	0.010
Occupation	Yes	458 (38.4)	194 (20.2)		
	No	735 (61.6)	766 (79.8)	83.30	<0.001
Current drinking	Yes	654 (40.8)	274 (30.9)		
	No	450 (59.2)	614 (69.1)	159.33	<0.001
Frequency of alcohol consumption		1.90±1.12	2.00±1.09	-1.20	0.229
Volume of alcohol consumption		2.09±1.31	1.70±1.06	4.45	<0.001
High-risk drinking	Yes	42 (6.4)	9 (3.3)		
	No	610 (93.6)	265 (96.7)	3.69	0.055
Depression	Yes	53 (4.4)	80 (8.3)		
	No	1,141 (95.6)	881 (91.7)	13.88	<0.001
Stress		2.03±0.68	2.26±0.72	-7.58	<0.001
Subjective health status		3.62±0.68	3.58±0.71	1.38	0.167
Subjective dental health		3.14±0.81	3.03±0.82	3.02	0.003
Sleep duration (hr)		7.49±1.27	6.48±1.31	18.12	<0.001
Unmet medical needs	Yes	123 (10.3)	227 (23.6)		
	No	1,071 (89.7)	735 (79.4)	69.25	<0.001

Values are presented as number (%) or mean±standard deviation.

**Table 2.** Relationships among variables

Variables			1	2	3	4	5	
							Yes	No
Pregnant	1. Stress	*r*	1.00					
	2. Subjective health status		-0.24^***^	1.00				
	3. Subjective dental health		-0.12^***^	0.27^***^	1.00			
	4. Sleep duration		-0.16^***^	0.08^***^	0.06	1.00		
	5. Depression							
	Yes		2.53±0.70	3.40±0.79	2.85±0.70			
	No		2.01±0.68	3.63±0.67	3.15±0.81			
	χ² or t		-5.47^***^	2.13^*^	3.11^*^			
	6. Unmet medical needs							
	Yes		2.31±0.74	3.47±0.73	2.85±0.81		12 (22.6)	111 (9.7)
	No		2.00±0.67	3.64±0.67	3.17±0.80		41 (77.4)	1,030 (90.3)
	χ² or t		-4.79^***^	2.43^*^	4.16^***^		9.14^*^	
Breastfeeding	1. Stress	*r*	1.00					
	2. Subjective health status		-0.21^***^	1.00				
	3. Subjective dental health		-0.12^***^	0.23^***^	1.00			
	4. Sleep duration		-0.18^***^	0.07^*^	0.03	1.00		
	5. Depression							
	Yes		2.81±0.73	3.29±0.75				
	No		2.21±0.70	3.61±0.70				
	χ² or t		-7.07^***^	3.69^***^				
	6. Unmet medical needs							
	Yes		2.53±0.73	3.36±0.72	2.75±0.80		40 (50.0)	187 (21.2)
	No		2.18±0.70	3.65±0.69	3.12±0.81		40 (50.0)	694 (78.8)
	χ² or t		-6.62^***^	5.26^***^	6.08^***^		33.66^***^	

Values are presented as number (%) or mean±standard deviation.

* p< 0.05,

*** p<0.001.
